# Does the availability of single cigarettes promote or inhibit cigarette consumption? Perceptions, prevalence and correlates of single cigarette use among adult Mexican smokers

**DOI:** 10.1136/tc.2008.029132

**Published:** 2009-11-17

**Authors:** J F Thrasher, V Villalobos, A Dorantes-Alonso, E Arillo-Santillán, K Michael Cummings, R O’Connor, G T Fong

**Affiliations:** 1 Department of Health Promotion, Education and Behavior, Arnold School of Public Health, University of South Carolina, USA; 2 Center for Population Health Research, National Institute of Public Health, Mexico; 3 Department of Health Behavior, Roswell Park Cancer Institute, USA; 4 Department of Psychology, University of Waterloo, Canada; 5 Ontario Institute for Cancer Research, Toronto, Ontario, Canada

## Abstract

**Background:**

Single cigarette use and its implications have rarely been studied among adults.

**Objective:**

To assess perceptions, prevalence and correlates of single cigarette purchase behaviour and its relation to harm reduction.

**Design:**

Focus group transcripts and cross-sectional data were analysed.

**Setting and participants:**

Focus groups among convenience samples of adult smokers in two Mexican cities and a population-based sample of 1079 adult smokers from the International Tobacco Control Policy Evaluation Project in four Mexican cities.

**Main outcome measures:**

Purchase of single cigarettes last time cigarettes were bought, frequency of purchasing single cigarettes in the previous month and intention to quit in the next 6 months.

**Results:**

Focus group data indicated that smokers bought single cigarettes as a harm reduction strategy. Survey data indicated that 38% of participants purchased single cigarettes in the last month and 10% purchased them the last time they bought cigarettes, with more frequent consumption among young adults and those with lower income. Purchasing single cigarettes was independently associated with the frequency of using single cigarettes to reduce consumption and, less consistently, with the frequency of being cued to smoke after seeing single cigarettes for sale. Using single cigarettes to reduce consumption was positively associated with quit intention, whereas being cued to smoke by single cigarettes was negatively associated with quit intention.

**Conclusions:**

Study results suggest that some adult Mexican smokers purchase single cigarettes as a method to limit, cut down on and even quit smoking. Nevertheless, promotion of the availability of single cigarettes as a harm reduction strategy could provide additional smoking cues that undermine quit attempts and promote youth smoking.

The Framework Convention for Tobacco Control (FCTC) states that the sale of single cigarettes should be prohibited, presumably because it increases affordability and accessibility of tobacco for minors.^1^ Research on this topic has mostly focused on the issue of youth access to cigarettes, including the prevalence of single cigarette purchases among youths^2^; “stings” to see if vendors will sell illegal single cigarettes to minors^3^ or adults^4^; assessment of the “peer market” for selling single cigarettes among adolescents in school^5^; and retailer interventions to reduce such the sale of single cigarettes.^6^ ^7^

The consumption of single cigarettes among adults is little studied. In the United States, the sale of single cigarettes may be most prevalent in neighbourhoods characterised by socioeconomic disadvantage, where such sales often occur in the unregulated, informal economy among street vendors.^8^ One survey of young adult inhabitants of a disadvantaged neighbourhood found that 77% had bought single cigarettes in the previous month, and their reasons ranged from factors that appeared to promote consumption (66% bought single cigarettes because of convenience and 59% bought them because of lower expense) and those that appeared to inhibit it (48% bought single cigarettes to cut down and 21% bought them to keep from getting addicted).^9^ It is unknown how these apparently contradictory tendencies play out among adult smokers in developing countries, where single cigarette sales may be more common than in high-income countries.

In Mexico, it has been illegal to sell single cigarettes since 1999.^10^ As in other countries,^2^ ^3^ ^4^ Mexican shop owners do not always comply with youth access laws.^11^ Furthermore, the illegal sale of single cigarettes appears prevalent. One Mexico City study of supermarkets, convenience stores, liquor stores, street vendors, news kiosks and small neighbourhood stores run out of private homes found that 58% of the stores surveyed sold single cigarettes.^10^ At one Mexican university, single cigarettes were widely sold on campus, facilitating the social exchange of cigarettes that is prevalent among youths and young adults.^12^ To better understand the role that single cigarettes have in Mexico, the present study examined focus group and survey data to assess the prevalence, perceptions and correlates of single cigarette use among adult Mexican smokers.

## Methodology

### Focus groups

#### Study sample

In preparation for the subsequent survey of smokers, in 2006, we conducted 10 focus groups with convenience samples of adult smokers in Cuernavaca and Mexico City, Mexico. Groups were conducted with students at two public universities (n = 3); among clerical, administrative and maintenance staff at a government hospital (n = 2) and at two public universities (n = 3); and among employees of the national gas and electric company (n = 2).

#### Focus group guide and analysis

The focus group guide aimed to gauge the meanings, contexts and experiences of smoking, as well as perceptions of existing and potential tobacco control policies. As part of the guide’s first section on sources and prices of cigarettes, participants were asked “How often do you purchase single cigarettes?” and “Does the availability of single cigarettes make you smoke more or less than you would otherwise?” All focus groups were audiotaped, transcribed and entered into the Atlas.ti qualitative data analysis program.^13^ Content analysis involved five stages^14^: reading the transcripts and developing a coding system to characterise content; applying codes to narrative segments; displaying similarly coded data in matrices with matrix rows containing relevant narrative segments; examination of data to discern the primary concepts and associations across focus groups and individual participants; interpretation of the data in light of the aims and the relevant literature.

### Survey

#### Study sample

Data were drawn from the 2006 administration of the International Tobacco Control Policy Evaluation Project in Mexico (ITC-Mexico), an international effort to understand tobacco policy impacts among cohorts of adult smokers in different countries.^15^ ^16^ ^17^ Sampling involved a multistage sampling scheme within selected cities (Mexico City, Guadalajara, Tijuana and Ciudad Juárez) to assemble a population-based representative cohort (see Thrasher *et al*^18^ for more detail on methods). Of the 4282 households that were approached, contact was established with the inhabitants of 2784 households (65%). Of the households contacted, 90% (n = 2499) were enumerated, yielding a household enumeration rate of 58%. Of the 1216 eligible smokers selected to participate from these enumerated households, interviews were conducted with 1079 (89%).

#### Measurement

##### Survey development

Survey questions went through four stages before being fielded. First, the 10 aforementioned focus groups were conducted in adult smokers to determine how they understood and talked about smoking.^19^ Second, previously validated questions used in other countries^17^ were put through a committee translation process, whereby four professional translators independently translated these questions and an adjudication meeting was held with the bilingual primary investigator to reach consensus on the best translation.^20^ Third, two rounds of cognitive interviewing were conducted with adult smokers to ensure adequate and equivalent comprehension of items.^21^ Fourth, a pilot survey was conducted in a sample of 100 people using the same protocol as the present study. Interviewer feedback from this field test indicated good understanding of the questionnaire.

##### Smoking behaviour

Participants were asked to indicate whether they bought single cigarettes, a cigarette pack or a carton of cigarettes at their last cigarette purchase. Responses were coded as purchasing either single cigarettes (1) or a pack or carton (0). Participants who bought single cigarettes were also asked how many single cigarettes they had bought and how much they paid per cigarette. The place of last cigarette purchase was also queried, and response options were combined to create meaningful groupings (that is, neighbourhood stores; convenience store or gas station; newsstand or street vendors; supermarket; and other). Self-reported smoking frequency was used to categorise respondents as either daily (0) or non-daily (1) smokers. A variable for recent quit behaviour was derived by determining whether the participant had attempted to quit in the previous year (1) or not (0). Participants were asked of their intentions to quit, and were categorised as intending to quit in the next 6 months (1) or not (0).

Participants were asked four additional questions about single cigarettes, with the previous month as the reference period: how often did they buy single cigarettes; how often did they buy single cigarettes to reduce the amount they smoke; how often did they see single cigarettes being sold; and how often did the feel cravings to smoke upon seeing single cigarettes being sold. Response options were never (0), sometimes (1), often (2), and very often (2). Owing to relatively small numbers for the response “very often,” the latter two categories were collapsed for analyses. People who responded “never” to the first question skipped out of the second question, but were recoded as “never” for the second question. Smokers are highly reactive to cues to smoke,^22^ and we derived a variable to capture the frequency of cravings to smoke as a result of seeing the sale of single cigarettes. To derive this measure we multiplied frequency of exposure (that is, seeing single cigarettes for sale) and frequency of cravings upon exposure (that is, cravings upon seeing single cigarettes sold) to get a 4-point ordinal scale. In bivariate and multivariate models, this indicator and the indicator of frequency of buying single cigarettes to reduce consumption were dummy coded, with the lowest frequency category as the reference group.

##### Sociodemographic variables

Respondents were asked to report their age, sex, highest educational level completed and monthly income (reported in Mexican pesos, which were approximately 10 pesos to $US1 at the time of data collection). Education and income were reclassified to the four categories that reflected the most uniform distribution possible (that is, less than middle school, middle school, high school or technical school, and more than high school; 0–3000 pesos, 3001–5000 pesos, 5001–8000 pesos and 8001 pesos or more per month), and dummy variables were created with the lowest level as the reference group.

#### Analysis

Analyses were conducted using Stata, version 8.0. Univariate descriptions of the study sample were calculated without adjustment; however, all other analyses adjusted for the design effect and sampling weights. A survey adjusted Pearson χ^2^ test was used to determine differences in the places where people bought their last cigarette and when comparing those who bought single cigarettes versus those who bought packs or cartons. Logistic regression was used when estimating bivariate and multivariate odds ratios relating study variables to buying single cigarettes at the last purchase and to intention to quit. Ordinal regression models were used to estimate the bivariate and multivariate adjusted associations between study variables and frequency of purchasing single cigarettes.

## Results

### Focus group results

#### Focus group sample characteristics

A total of 75 adult smokers participated, and each focus group comprised 5–12 people. The average age of all participants was 33, ranging from 19–70 years old; however, the average age was 21 across the three groups with university students, whereas it was 39 across the other groups. Males and females were equally represented (n = 38 and 37, respectively). Forty-eight per cent of participants smoked less than five cigarettes a day and 23% smoked more than 10 cigarettes a day. Thirty-nine per cent said that had attempted to quit in the previous year and 37% said that they had never tried to quit.

#### Buying single cigarettes to control consumption

One or more participants in each focus group said that they purchased single cigarettes and described how they purchased single cigarettes as a method of quitting, cutting down or keeping from smoking too many cigarettes. One person typified this perspective when she stated “I used to buy packs, but having the pack, I would smoke one after another. So I began to buy singles …to smoke less.” In explaining how smoking single cigarettes inhibited consumption, participants drew attention to the additional cost of single cigarettes per cigarette as well as the extra effort involved in finding single cigarettes to purchase compared to having a pack on hand. For example, one participant stated:

…if you have a pack…you don’t take a break between cigarettes, [you smoke] one after another. However, when you buy singles, between costing more and not being as accessible, even when you want a cigarette, and you can’t find it everywhere, you have to look for where they sell a single or for someone who will give you one.

One participant also described how the high unit cost of single cigarettes could caused her to revert to buying packs after trying to use single cigarettes as a method to keep consumption down:

When I figured out that I couldn’t quit smoking, I began to buy singles, trying to not have the pack and smoke a lot. But I was doing it, and yeah I felt like I was paying more, I mean, to pay two pesos per cigarette and when you check how much it costs, it’s double what a pack costs, which I didn’t like, so I don’t buy singles any more.

Hence, the additional cost of purchasing single cigarettes may cause some people to go back to packs over the long term, with single cigarettes serving to keep people smoking until they finally give in and purchase packs to sustain their consumption more cheaply.

#### Seeing single cigarettes as a cue to smoke

Some, although fewer, participants described the widespread sale of single cigarettes as cuing them to smoke. One participant described how the cueing was more apparent to him when he tried to cut down on cigarettes:

When I do not want to smoke, I look for a way to keep from having cigarettes around, but when I go a few hours, like maybe four hours, without smoking, then if I see it then I buy it, including at stoplights, where kids sell [singles].

Participants did not spontaneously describe the more mundane cueing to smoke that the availability of single cigarettes may provide, even when smokers are not trying to cut down or quit. Overall, these results suggested that the availability of single cigarettes provokes conflicting tendencies in promoting and inhibiting consumption.

### Survey results

#### Survey sample characteristics

The sample comprised 61% males and had an average age of 38.6 years (see table 1). Seventy-nine per cent of participants were daily smokers, 26% had tried to quit in the previous year, and 16% intended to quit in the next 6 months. Ninety per cent of the population bought a cigarette pack at their last cigarette purchase, whereas 9% bought single cigarettes and less than 1% (n = 4) bought cartons. Among those who bought single cigarettes at their last purchase, 73% bought between one and three cigarettes. The average cost per single was 1.68 pesos (approximately $0.15), which was almost double the unit cost of a cigarette when purchased in a pack of 20. Most smokers did not purchase single cigarettes in the previous month (62%) or experience cravings to smoke after seeing the sale of single cigarettes (60%). Seventy-five per cent of participants did not purchase single cigarettes to control their consumption in the previous month.

**Table 1:** Sample characteristics

Sample characteristics	% (n)
Sex	
Female	39% (422)
Male	61% (657)
Age	
18–24	16% (170)
25–39	40% (432)
40–54	31% (335)
55+	13% (142)
Education	
< Middle school	28% (290)
Middle school	27% (281)
High school	30% (312)
> High school	14% (144)
Income (pesos/month)	
0–3000	25% (248)
3001–5000	31% (320)
5001–8000	25% (256)
8001 or more	16% (162)
Smoking status	
Non-daily smoker	21% (231)
Daily smoker	79% (848)
Quit attempt in previous year	26% (278)
Intention to quit in next 6 months	16% (171)
Last purchase	
Pack	90% (963)
Single cigarettes	9% (97)
Carton	0% (4)
Frequency of purchasing single cigarettes in the last month	
Never	62% (667)
Once in a while	27% (294)
Often	7% (72)
Very often	4% (40)
Frequency of purchasing single cigarettes to reduce consumption	
Never	76% (816)
Once in a while	15% (163)
Often/very often	9% (91)
Frequency of cravings to smoke after seeing single cigarettes	
None	60% (624)
Low	13% (135)
Middle	17% (126)
High	11% (110)

#### Location of last cigarette purchase

When assessing the places where people bought their last cigarettes, we compared people who bought single cigarettes with those who bought packs and cartons, combining the last two categories owing to the low number of people who bought cartons (n = 4). An omnibus Pearson χ^2^ test, adjusted for the design effect, indicated a significant difference in purchase venues by cigarette type (p = 0.003). The vast majority of smokers bought their cigarettes in small neighbourhood stores, whether they bought single cigarettes (83.0%) or packs (78.5%). Convenience stores and gas stations were the second most prevalent source for purchasing packs (13.3%), although some smokers who bought single cigarettes also bought them there (4.7%). Newsstands and street vendors also served as sources for last pack purchase (2.1%), as well as for single cigarette purchase (5%). Study participants only bought packs (3.4%), not single cigarettes, from supermarkets. When survey adjusted Pearson χ^2^ tests were conducted to determine differences in prevalence of a particular purchase site, the only statistically significant difference was the higher prevalence of purchasing packs versus single cigarettes at convenience stores and gas stations.

#### Correlates of purchasing single cigarettes

The survey adjusted prevalence of buying single cigarettes at the last cigarette purchase was 10%. Bivariate logistic regression models were run to assess characteristics associated with this purchase (see table 2). Younger age, less education, lower income, non-daily smoking, having attempted to quit in the last year, greater intentions to quit, more frequently buying single cigarettes to reduce the amount smoked, and more frequent cues to smoke when seeing single cigarettes for sale were all associated with buying single cigarettes at last purchase. In the multivariate logistic regression model (see table 2), the likelihood of purchasing single cigarettes was positively associated with lower income levels, smoking less regularly (that is, non-daily vs daily smoker), intending to quit, frequency of smoking single cigarettes to control consumption and frequency of cues to smoke after seeing single cigarettes for sale. The strongest of these independent correlates was having often or very often bought single cigarettes to reduce consumption, which was associated with a 16 times greater likelihood of buying single cigarettes at the last purchase compared to those who did not buy single cigarettes to reduce consumption (OR_often/very often vs none_ 16.08; 95% CI 6.30 to 41.04).

**Table 2:** Bivariate and multivariate adjusted logistic models for buying single cigarettes at last cigarette purchase

Independent variables		Bivariate OR (95% CI)	Multivariate OR (95% CI)
Sex			
Female		1	1
Male		0.83 (0.48 to 1.44)	0.76 (0.42 to 1.39)
Age			
18–24		1	1
25–39		0.59 (0.29 to 1.20)	1.01 (0.43 to 2.37)
40–54		0.27 (0.12 to 0.64)†	0.47 (0.16 to 1.39)
55+		0.34 (0.13 to 0.93)*	0.77 (0.26 to 2.26)
Education			
< Middle school		1	1
Middle school		0.98 (0.47 to 2.07)	1.02 (0.38 to 2.73)
High school		0.61 (0.28 to 1.31)	0.75 (0.33 to 1.71)
> High school		0.26 (0.08 to 0.90)*	0.29 (0.08 to 1.13)
Income (pesos/month)			
0–3000		1	1
3001–5000		0.52 (0.26 to 1.02)	0.41 (0.17 to 0.97)*
5001–8000		0.45 (0.21 to 0.98)*	0.44 (0.17 to 1.12)
8001 or more		0.41 (0.26 to 0.65)§	0.47 (0.25 to 0.88)*
Regularity of smoking			
Daily		1	1
Non-daily		2.52 (1.48 to 4.26)‡	2.52 (1.31 to 4.81)†
Quit attempt in last year			
No		1	1
Yes		1.57 (0.97 to 2.55)	0.91 (0.40 to 2.05)
Intention to quit in next 6 months			
No		1	1
Yes		3.00 (1.56 to 5.78)‡	2.29 (1.17 to 4.48)*
Frequency of buying single cigarettes to reduce consumption, last month			
Never		1	1
Once in a while		6.72 (3.31 to 13.65)§	2.63 (0.90 to 7.71)
Often/very often		19.23 (8.28 to 44.69)§	16.08 (6.30 to 41.04)§
Cues to smoke after seeing single cigarettes for sale, last month			
None		1	1
Low		4.00 (1.61 to 9.93)†	2.92 (1.08 to 7.91)*
Middle		2.97 (1.46 to 6.04)†	2.19 (0.92 to 5.20)
High		3.64 (1.51 to 8.77)†	1.91 (0.68 to 5.39)

*p<0.05; †p<0.01; ‡p<0.001; §p<0.0001.

Table 3 shows the bivariate and multivariate adjusted coefficients from ordinal regression models that estimate the association between study variables and self-reported frequency of purchasing single cigarettes in the last month. Younger age, lower income, having a recent quit attempt, quit intentions, frequency of buying single cigarettes to reduce consumption and frequency of cravings upon seeing single cigarettes were all associated with the frequency of purchasing single cigarettes in the previous month. In the multivariate model, the only statistically significant independent associations were with younger age, frequency of buying single cigarettes to reduce consumption and the frequency of craving single cigarettes upon seeing them sold. As with the analysis of last purchase, the strongest correlate appeared to be the frequency of buying single cigarettes to reduce consumption.

**Table 3:** Bivariate and multivariate adjusted ordinal regression models for frequency of purchasing single cigarettes in last month

Independent variables		Bivariate OR (95% CI)	Multivariate OR (95% CI)
Sex			
Female		–	–
Male		0.107 (0.160)	0.209 (0.210)
Age			
18–24		–	–
25–39		−0.947 (0.201)§	−0.543 (0.309)
40–54		−1.270 (0.229)§	−0.891 (0.337)*
55+		−1.925 (0.311)§	−1.393 (0.368)‡
Education			
< Middle school		–	–
Middle school		0.230 (0.237)	0.207 (0.206)
High school		−0.371 (0.234)	−0.058 (0.238)
> High school		−0.129 (0.316)	−0.204 (0.300)
Income (pesos/month)			
0–3000		–	–
3001–5000		−0.110 (0.209)	−0.366 (0.242)
5001–8000		−0.580 (0.209)†	−0.416 (0.265)
8001 or more		−0.267 (0.097)*	−0.198 (0.107)
Regularity of smoking			
Daily		–	–
Non-daily		0.325 (0.230)	0.070 (0.236)
Quit attempt in last year			
No		–	–
Yes		0.801 (0.183)§	0.344 (0.231)
Intention to quit in next 6 months			
No		–	–
Yes		0.525 (0.210)*	0.199 (0.285)
Frequency of buying single cigarettes to reduce consumption, last month			
Never		–	–
Once in a while		3.633 (0.267)§	3.077 (0.300)§
Often/very often		6.173 (0.553)§	5.854 (0.583)§
Cues to smoke after seeing single cigarettes for sale, last month			
None		–	–
Low		1.401 (0.272)§	0.815 (0.312)*
Middle		1.745 (0.233)§	1.428 (0.259)§
High		1.918 (0.309)§	1.355 (0.293)§

*p<0.05; †p<0.01; ‡p<0.001; §p<0.0001.

#### Correlates of intention to quit

Logistic models were estimated regressing intention to quit in the next 6 months on study variables (table 4). In bivariate models, non-daily smokers were more likely than daily smokers to intend to quit (OR = 1.75) and those who most frequently bought single cigarettes in the last month to reduce consumption were more likely than those who did not to intend to quit (OR = 3.20). In multivariate models, those who most frequently purchased single cigarettes to reduce consumption remained more likely than those who did not to intend to quit (OR = 3.71), and two variables that were unassociated in bivariate models became associated with quit intentions in the multivariate model: males were less likely than females (OR = 0.62) and those who experienced the most frequent urges to smoke upon seeing single cigarettes were less likely than those who did not experience these urges (OR = 0.40) to intend to quit.

**Table 4:** Bivariate and multivariate logistic models of intention to quit in the next six months

Independent variables		Bivariate OR (95% CI)	Multivariate OR (95% CI)
Sex			
Female		1	1
Male		0.74 (0.54 to 1.01)	0.62 (0.43 to 0.90)*
Age			
18–24		1	1
25–39		1.44 (0.74 to 2.82)	1.55 (0.88 to 2.72)
40–54		0.85 (0.38 to 1.92)	0.97 (0.47 to 1.98)
55+		1.07 (0.44 to 2.61)	0.95 (0.36 to 2.48)
Education			
< Middle school		1	1
Middle school		1.36 (0.74 to 2.51)	1.44 (0.74 to 2.80)
High school		1.41 (0.70 to 2.81)	1.59 (0.70 to 3.56)
> High school		0.73 (0.30 to 1.76)	0.82 (0.29 to 2.33)
Income (pesos/month)			
0–3000		1	1
3001–5000		1.03 (0.63 to 1.67)	0.76 (0.45 to 1.30)
5001–8000		0.92 (0.42 to 2.01)	0.89 (0.36 to 2.20)
8001 or more		0.83 (0.65 to 1.06)	0.85 (0.64 to 1.12)
Smoking regularity			
Daily		1	1
Non-daily		1.75 (1.02 to 3.02)*	1.76 (0.94 to 3.29)
Quit attempt in last year			
No		1	1
Yes		1.19 (0.79 to 1.80)	1.17 (0.74 to 1.85)
Frequency of buying single cigarettes to reduce consumption, last month			
None		1	1
Once in a while		0.95 (0.56 to 1.61)	1.00 (0.57 to 1.77)
Often/very often		3.20 (1.74 to 5.86)§	3.71 (2.13 to 6.48)§
Cues to smoke after seeing single cigarettes for sale, last month			
None		1	1
Low		1.27 (0.70 to 2.30)	1.12 (0.60 to 2.08)
Middle		1.33 (0.78 to 2.29)	1.11 (0.61 to 2.01)
High		0.55 (0.26 to 1.16)	0.40 (0.19 to 0.85)*

*p<0.05; †p<0.01; ‡p<0.001; §p<0.0001.

## Discussion

Our results suggest that adult Mexican smokers buy single cigarettes with some regularity. Participants in every focus group purchased single cigarettes. Survey adjusted prevalence of purchased single cigarettes in the last month was 38% and the prevalence of buying single cigarettes at the last cigarette purchase was 10%. The only other published attempt to estimate prevalence of single cigarette use comes from a convenience sample of young US adults in disadvantaged areas, among whom 77% has purchased single cigarettes in the previous month.^9^ The lower prevalence of single cigarette use found in our sample is probably because of its population-based character. Nevertheless, our results are consistent with the notion that single cigarette use in Mexico is concentrated among younger smokers and smokers from lower income groups. In multivariate models, younger smokers more frequently purchased single cigarettes than older smokers, and there appeared to be a threshold effect for income, where the three highest income groups had a similarly decreased likelihood of having bought single cigarettes at the last cigarette purchase compared to the lowest income group. These findings support the notion that the availability of single cigarettes may help facilitate the early stages of nicotine addiction among young people and may keep disadvantaged groups smoking, even if it is at a lower intensity. Our data indicate that consumption of single cigarettes in Mexico is not limited to these populations, however.

The places where Mexican smokers buy single cigarettes are generally the same as those where they obtain cigarette packs, with approximately 80% of cigarette sales of either packs or single cigarettes take place in neighbourhood stores. Such stores often operate within the informal economy, which the World Bank estimates to account for 20% to 57% of all jobs in Mexico.^23^ The informal economy generally lies outside of the formal regulatory processes that aim to ensure implementation of commercial laws, including those that have made the sale of single cigarettes illegal.^24^ Location within the formal economy may decrease illegal single cigarette sales (no participant reported purchasing single cigarettes from supermarkets, for example); however, formal economic sector sales do not always follow the law, as has been found in previous research^10^ and was confirmed in our study’s finding that some smokers purchased their single cigarettes at convenience stores and gas stations. In countries like Mexico, the overall difficulty of effectively implementing a ban on single cigarette sales raises questions about prioritising this policy.

Given the difficulty of single cigarette bans, our results also provide heartening evidence of the possible harm reduction benefits of the availability of single cigarettes. Smokers in our focus groups indicated that the higher relative cost per cigarette and lower accessibility of single cigarettes compared to pack cigarettes makes the consumption of single cigarettes a viable strategy for limiting consumption and even helping to quit. Our survey and focus group data were consistent in finding that single cigarettes cost approximately double the unit price of a cigarette when bought in a pack of 20. Smokers are price sensitive across countries,^25^ including Mexico,^26^ and this sensitivity probably helps to explain why purchasing single cigarettes can inhibit consumption. The availability of cheaper cigarettes by the pack may nevertheless stifle attempts to use this method among those who are concerned about cost, as was recounted by one focus group participant.

Our survey results suggest that smokers who say that they use single cigarettes to control consumption are not only more likely to consume single cigarettes but they are also more likely to intend to quit than smokers who do not purchase single cigarettes to control consumption. Determining whether such quit intentions and accompanying use of single cigarettes translates into actual quit behaviour will require longitudinal analysis. Nevertheless, we expect that the relation between quit intention and quit behaviour will apply among Mexican smokers. If this holds true, then the greater price and extra effort necessary to find single cigarettes for sale may contribute to lower levels of consumption and, potentially provide smokers with a harm reduction strategy that has heretofore not been considered in debates on that issue.^27^ Indeed, young adults from a disadvantaged area in the US also indicated that they used single cigarettes as a harm reduction strategy.^9^ The harm reduction method may be particularly relevant to the case of Mexico, where even daily smokers smoke 6.7 cigarettes a day,^28^ which is a lower smoking intensity than in other countries.^29^

Our results also suggest that the potential public health impact of the availability of single cigarettes for harm reduction may be at least partly offset by the pro-smoking cues that their availability provides to smokers in general. Our index of the frequency of cravings to smoke upon seeing single cigarettes for sale was positively associated with single cigarette consumption, although the relation was weaker than for the frequency of smoking single cigarettes to reduce consumption. Those who experienced the most frequent cues to smoke from seeing single cigarettes for sale were less likely to intend to quit than those who did not experience such cues, whether because they reported not seeing single cigarettes for sale or, upon seeing them, they did not experience cravings. The population of smokers who react most strongly to the cues provided by single cigarettes may also be more receptive to other environmental cues to smoke, such as advertising, ash trays and cigarette package displays.^30^ If these other cues are not reduced or eliminated, then eliminating the cue of single cigarettes from their environment may have only a marginal effect on smokers’ consumption.

Research on cues to smoke has been conducted almost exclusively in laboratory settings and has only recently been studied in natural settings.^30^ To our knowledge, our effort to use surveys to capture exposure to cues and reactivity towards them is relatively novel. The associations we found between our measure and variables of interest provide evidence of construct validity. Although we used cognitive interviewing to confirm adequate comprehension of the measures we used, our measure may nevertheless suffer from biases associated with self-report, including potential under-reporting of a cue like this that could operate at a relatively unconscious level. Future research should further assess the reliability and validity of our measure, as well as other measurement approaches, in order to better understand how cueing works outside of laboratory settings.

**What this paper adds**

- Previous research on single cigarette vending and use has been focused on its potential to promote youth smoking. Single use among adults has received less attention, and some smokers have reported that they purchase more costly and relatively less accessible single cigarettes as a harm reduction strategy.
- The results from this study suggest that the availability of single cigarettes may provide adult smokers in Mexico with a viable strategy for cutting down and even quitting smoking. However, some smokers report that seeing single cigarettes cues smoking urges, thereby potentially undermining any beneficial effects associated with their availability.

There are also a number of other limitations to this study. The cross-sectional nature of the data precludes determination of causality for time varying characteristics. Longitudinal data analysis will be necessary to more adequately determine whether those who consume single cigarettes actually reduce consumption or quit at higher rates than those who do not. Relapse behaviour also should be examined to determine whether some ex-smokers may resume smoking because of the cues provided by the availability of single cigarettes in their environment. The results may not generalise to smokers in rural areas or other cities in Mexico. Furthermore, the moderate participation rate may also mean that the results do not generalise to all adult smokers in the cities where data were collected. Similar studies should be conducted in other countries, particularly where smokers have heavier smoking habits, as the relatively light smoking habit among Mexicans may limit these conclusions to Mexico.

In summary, this study provides evidence that the relatively widespread availability of single cigarettes and the practice of single cigarette consumption in Mexico may facilitate cutting down or even quitting, perhaps providing a harm reduction strategy that has heretofore not been considered. Nevertheless, our study also suggests that the availability of single cigarettes may promote smoking. Further studies should more squarely focus on this issue, as well as whether single cigarette availability promotes youth smoking. Younger smokers were more likely to more frequently purchase single cigarettes, providing some support for the idea of a gateway effect. Hence, single cigarette availability and use may facilitate heavier use.
